# Successful surgery of a giant gallbladder with multiple stones accompanied by symptoms of acute cholecystitis and nursing care: a case report

**DOI:** 10.1097/RC9.0000000000000674

**Published:** 2026-07-17

**Authors:** Zahra Eghbali, Hamed Tavan

**Affiliations:** a Department of Surgery, School of Medicine, Shahid Beheshti University of Medical Science, Tehran, Iran; b Student Research Committee, Faculty of Nursing and Midwifery, Ilam University of Medical Sciences, Ilam, Iran

**Keywords:** acute cholecystitis, gallstones, giant gallbladder, nursing care, surgery

## Abstract

**Introduction:**

Acute cholecystitis is a common surgical condition and the most frequent cause of right upper quadrant (RUQ) abdominal pain, accounting for 3–10% of all abdominal pain cases. This case report describes the successful surgical management of a giant gallbladder with multiple stones, presenting with symptoms of acute cholecystitis, along with the associated nursing care.

**Case presentation:**

A 42-year-old man with a history of methadone and alcohol use presented with RUQ abdominal pain. The diagnosis was confirmed via ultrasound, laboratory tests, clinical history, and physical examination by a general surgeon and radiologist. Upon admission, the patient’s visual analog scale (VAS) pain score was 7 out of 10, which decreased to 5 preoperatively and to 2 postoperatively. Vital signs remained normal throughout the hospitalization. Due to the unavailability of laparoscopic equipment, an open cholecystectomy was performed via a Kocher incision. Intraoperatively, a giant gallbladder measuring approximately 20 cm × 8 cm was identified. A chevron incision was subsequently made along the Kocher line. The surgery was completed successfully.

**Discussion and conclusion:**

Accurate diagnosis and timely surgical intervention are critical in gallbladder pathology, and structured postoperative nursing care is highly recommended.

## Introduction

Gallstones are a common biliary tract disorder for which cholecystectomy has been the standard treatment since 1882. In Western countries, approximately 10–15% of the adult population is affected by gallstones. Symptoms may include intermittent right upper quadrant (RUQ) pain, nausea, and vomiting. Acute cholecystitis is one of the most common surgical conditions and accounts for 3–10% of all cases of RUQ abdominal pain^[^1^]^. In the United States, it affects over 20 million people annually, incurring substantial healthcare costs^[^2^]^. Classic symptoms include RUQ pain, fever, and leukocytosis^[^3^]^. Timely and accurate diagnosis is essential to initiate treatment and reduce mortality; however, even after a thorough history and physical examination, the diagnosis often remains ambiguous, necessitating more advanced and costly imaging^[^4^]^. RUQ ultrasound is considered the gold standard for diagnosing cholecystitis^[^5^]^. Surgery remains the definitive treatment.

HIGHLIGHTS

- Appropriate diagnosis and timely surgical intervention are crucial in gallbladder surgery.
- Nursing care is highly recommended.
- Diagnosis was confirmed through ultrasound, blood tests, and history.
- Physical examination was performed by a general surgeon and a radiologist. Successful surgery was subsequently performed.

Surgical options include open and laparoscopic cholecystectomy, with the latter being more common today. Open surgery carries a risk of abdominal wall damage, whereas laparoscopic surgery, though minimally invasive, may result in bile duct injury, acute biliary obstruction, or extrahepatic duct rupture. Nevertheless, laparoscopic cholecystectomy offers smaller incisions and shorter hospital stays. The reported incidence of bile duct injury is 1–3% in open cholecystectomy and 4–6% in laparoscopic cholecystectomy^[^6^]^. Another study also stated that the rate of bile duct injury increased with the laparoscopic method; however, laparoscopic bile duct injuries are more common but less dangerous compared to the open method, which is why it is preferred ^[^7^]^.

Patients with acute cholecystitis require care from nurses knowledgeable in diagnostic and therapeutic interventions. Nursing responsibilities include preoperative preparation, postoperative care, complication management, and discharge education.

This report describes the successful surgical management of a giant gallbladder with multiple stones, presenting with acute cholecystitis, along with the nursing care provided.

## Case presentation

A 42-year-old male patient with a history of methadone and alcohol dependence presented with RUQ abdominal pain persisting from morning to night. Abdominal examination revealed mild distension, tenderness, and rebound tenderness in the epigastric region and RUQ. Initial chest and abdominal radiographs showed no free air or other pathological findings. Initial laboratory results were as follows: WBC = 15 000/µL, neutrophils = 81%, liver function tests = normal, amylase = 100 U/L, lipase = 58 U/L. Abdominal and pelvic ultrasound revealed grade 1 fatty liver and normal intra- and extrahepatic bile duct diameters. The gallbladder was enlarged, measuring 10 cm, and contained sludge and multiple stones with an average diameter of 9 mm, suggestive of acute cholecystitis.

Because laparoscopic equipment was unavailable, an open cholecystectomy was performed via a Kocher incision. A giant gallbladder measuring approximately 20 cm × 8 cm was identified. A chevron incision (a surgical incision beginning at the midline below the axilla and extending across the abdomen, commonly used in liver and upper abdominal surgeries, including gallbladder procedures) was made along the Kocher line.

**Figure 1.**
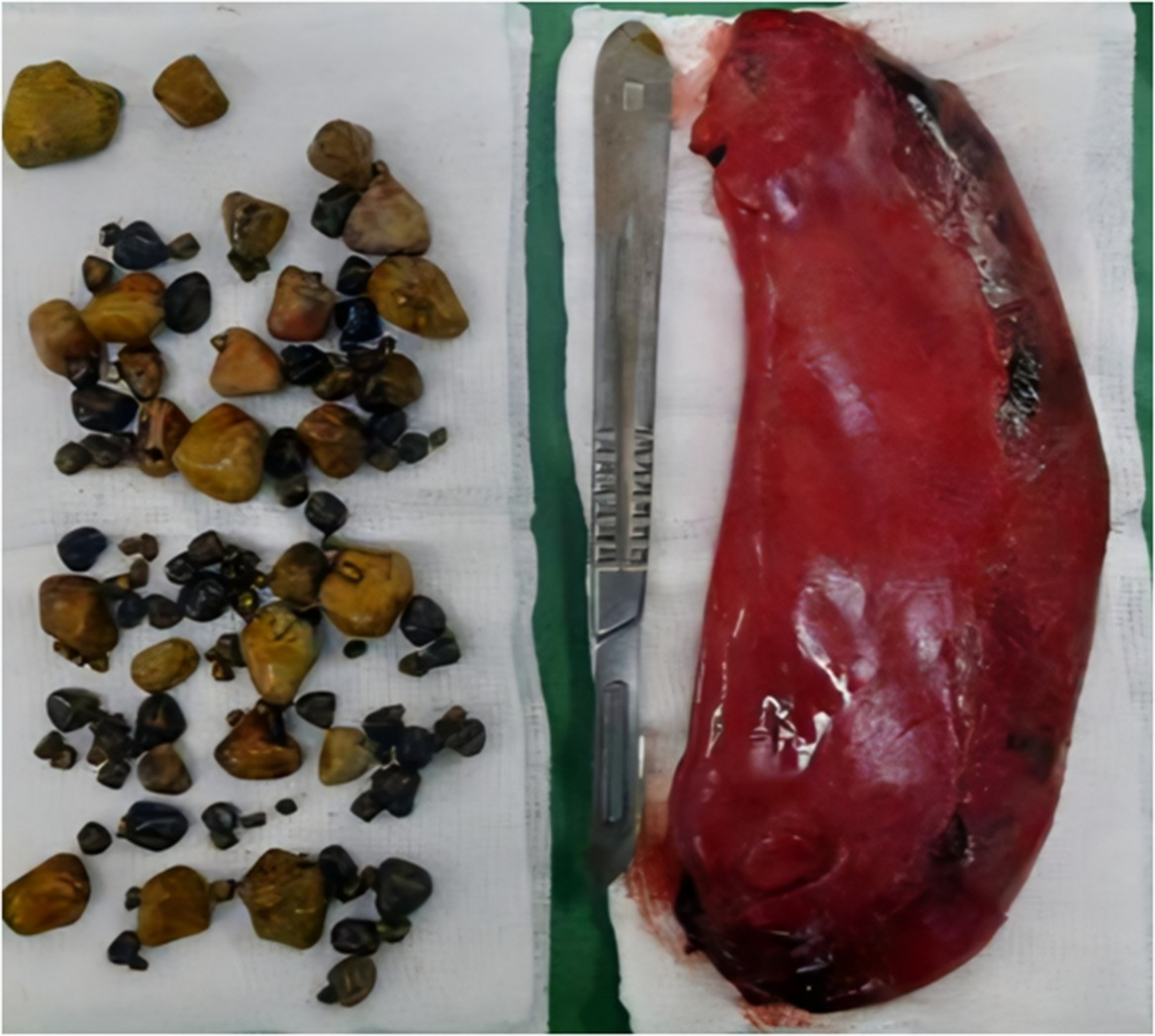
Intraoperative view. Successful surgical management of a giant gallbladder with multiple stones, presenting with symptoms of acute cholecystitis.

The gallbladder fundus was punctured, and approximately 500 cc of bile and stones were aspirated. The gallbladder was then dissected from the liver bed. The cystic duct and cystic artery were individually double-ligated. The specimen was sent for pathological examination. A Hemovac drain was placed; it produced no significant output and was subsequently removed. The patient was discharged in good general condition the following day. Pathology results are presented in Table 1, and an intraoperative image is shown in Figure 1.

**Table 1:** Pathology results.

Section	Description
Gross description	Received specimen in formalin consists of gallbladder measuring 18 cm in length and 6 cm in maximum diameter. External surface is irregular and gray brown. Mucosa is creamy brown, and wall thickness is 1.3 cm maximally. Multiple yellow brown stones are seen in the lumen. SOS = 1/1, EMBEDDED: 1%.
Microscopic description	Microscopic examination shows gallbladder wall structure with infiltration of acute and chronic inflammatory cells, hemorrhage, necrosis, and fibrosis. No neoplastic tissue is identified in multiple sections of the representative specimen.
Diagnosis	Gallbladder, cholecystectomy: acute on chronic cholecystitis with cholelithiasis.

Although the standard of care is laparoscopic cholecystectomy, it was not available in this setting. The procedure was, therefore, performed via a Kocher incision, which was converted to a chevron incision due to the large gallbladder size. This technique may be considered in hospitals lacking laparoscopic equipment.

**Vital signs and pain**: The patient’s vital signs – including blood pressure, respiratory rate, pulse rate, and temperature – remained normal upon admission, preoperatively, and postoperatively. However, the patient’s abdominal pain score on the visual analog scale was 7 upon admission, 5 preoperatively, and 2 postoperatively.

This paper has been reported in line with the SCARE 2025 criteria^[^8^]^.

## Discussion

In a study by Gao *et al* titled “Laparoscopic cholecystectomy for giant gallbladder: A case report,” the authors noted that an overly distended gallbladder is typically observed in cases of distal biliary obstruction due to malignancy. The gallbladder may also become enlarged and distended during obstruction of the cystic duct or gallbladder neck by gallstones^[^1^]^.

Terashi *et al*, in “Giant gallbladder cyst with acute cholecystitis: A Case Report,” reported that giant gallbladders are rare, with very few documented cases, and no clinical or histological definition has been established. Histological examination revealed swollen gallbladder specimens with cystic lesions on the external wall, accompanied by wall thickening and inflammation. The cystic lesion indicated gallbladder duplication, gallbladder diverticulum, or extension of the Rokitansky–Aschoff sinuses^[^2^]^.

Cheng *et al*, in “A case of H-type double gallbladder anomaly: A case report,” concluded that biliary system anomalies, such as double gallbladders, are rare congenital conditions that pose significant diagnostic challenges. The Boyden classification, particularly the H-type anomaly, provides critical insight into these variations. Failure to diagnose these anomalies preoperatively increases the risk of surgical complications, making early identification crucial for surgical planning^[^3^]^.

## Nursing care in gallbladder surgery and summary of post-cholecystectomy care

Following gallbladder surgery, adherence to specific care measures is essential for rapid recovery and the prevention of complications. Key measures include a low-fat diet, avoidance of heavy foods, adequate hydration, avoidance of heavy lifting and strenuous activity, pain management with prescribed medications, proper wound care, and regular medical follow-ups. By strictly observing these points, patients can quickly return to daily life and prevent potential complications^[^9–11^]^.

## Conclusion

Accurate diagnosis and timely surgical intervention are critical in gallbladder surgery, and structured postoperative nursing care is highly recommended.

## Suggestions and recommendations

The findings of this report may enhance knowledge and attitudes regarding gallbladder surgery. Educating caregivers about symptoms can significantly reduce subsequent disease-related risks.

## Data Availability

Not applicable.
